# Keynote Address (November 2016): Zika Virus Disease in the Americas: A Storm in the Making

**DOI:** 10.4269/ajtmh.17-0207

**Published:** 2017-05-01

**Authors:** Carissa Etienne, Thais dos Santos, Marcos A. Espinal

**Affiliations:** 1 Director, Pan American Health Organization, Washington, District of Columbia; 2 Communicable Diseases and Health Analysis Department, Pan American Health Organization, Washington, District of Columbia

## Abstract

More than 700,000 cases of Zika virus (ZIKAV) disease have been officially reported to the Pan American Health Organization (PAHO) from 48 countries and territories of the Americas. The response led by the PAHO and partners suggests major lessons of this outbreak. A seemingly innocuous pathogen became the new villain, causing fear, economic losses and, most importantly, debilitating birth defects and neurological problems, reaffirming the well-known war principle of never to underestimate one's opponent. The ZIKAV tested public health capacities under the International Health Regulations, highlighting the need for continued investment in health security. Last but not least, the lack of appropriate tools was another reminder of the pressing need for innovative solutions to persistent problems. Latin America and the Caribbean have approximately 500 million persons living in areas at risk for transmission of ZIKAV. The fight against ZIKAV is not a 100-m race, but rather a marathon in which science and public health need to work hand in hand for the benefit of our peoples.

## Background

In May 2015, the first case of Zika virus (ZIKAV) disease was diagnosed in the continental region of the Americas, in the State of Bahia, Brazil. This confirmation came following months of detections of clusters of rash illness in the northeast of Brazil.^1^ Soon after the detection of cases of ZIKAV, clinicians in the areas of virus circulation also detected a marked increase in neurological disorders, such as Guillain–Barre syndrome (GBS).^2^ This was followed by reports from the States of Pernambuco, Paraiba, and Rio Grande do Norte of a perceived increase in cases of microcephaly. There was also compelling evidence that many of the mothers of the children with microcephaly reported experiencing a febrile rash during their pregnancies.^3^ Following notification on the possible transmission of ZIKAV in the northeast of Brazil, the Pan American Health Organization (PAHO) issued an epidemiological alert to reinforce the recommendations made previously regarding *Aedes aegypti*—also the vector of yellow fever, chikungunya, and dengue fever viruses—for leveraging existing surveillance systems for dengue and chikungunya to increase their sensitivity to detect possible cases of ZIKAV infection.^4^

Considering the strong temporal and spatial association between infection with ZIKAV and a rise in detected cases of congenital malformations and neurological complications, on February 1, 2016, the World Health Organization (WHO) Director General declared that the ZIKAV event met the criteria of a Public Health Emergency of International Concern, calling for urgent international coordination and collaboration to better understand the full impact of the virus. As of February 2017, 48 countries and territories in the region of the Americas have confirmed autochthonous, vector-borne transmission of ZIKAV. Among these, 20 countries and territories have reported increases in the detection of GBS and/or laboratory confirmation of ZIKAV in at least one GBS case, and 23 countries and territories have reported cases of congenital syndrome associated with ZIKAV infection.^5^

**Figure 1.**
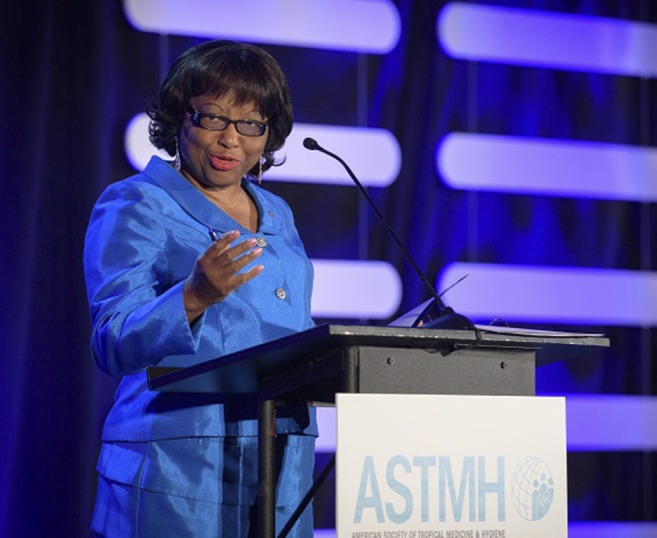
Carissa Etienne, MBBS, MSc, Director of PAHO, delivers the keynote address at the ASTMH 2016 Annual Meeting in Atlanta.

## Never Underestimate your Opponent

The Americas had the unfortunate distinction of being a testing ground for ZIKAV infections. For more than 50 years, ZIKAV remained a virtually silent, seemingly innocuous pathogen, causing only occasional outbreaks that had no major consequences. Then it became the new villain in this region, causing fear, economic losses and, most importantly, marking the lives of children and families affected by debilitating birth defects. Given the magnitude of the preliminary findings, an incident management structure was established in PAHO to provide timely guidance to Member States and to maximize the efficiency of the PAHO response. PAHO coordinated more than 80 expert missions to 30 countries affected by ZIKAV. These missions brought together neurologists, neonatologists, obstetricians, epidemiologists, virologists, and specialists in research and health services organization to bring the most up-to-date knowledge to countries in the response to this epidemic. Training and in-country activities were supported in every aspect of the response including clinical management, laboratory diagnosis, risk communication, and vector control. In addition, efforts were invested in the development of a Regional Research Agenda that identifies current gaps in knowledge and marshals partners to accelerate efforts to generate evidence to mitigate the impact of the virus.

Countries and territories of the Americas have officially reported 753,703 cases of ZIKAV disease as of March 2, 2017.^6^ It is not surprising that an outbreak of this magnitude has increased our knowledge of the virus and expanded the observed clinical spectrum of the disease. Although ZIKAV was first identified in 1947, the current outbreak has generated more knowledge about this pathogen than all of the previous outbreaks combined. A search of PubMed yielded 112 journal articles mentioning ZIKAV from 1952 until the end of 2014. This is inclusive of the outbreaks in The Federated States of Micronesia (Yap) in 2007 and in French Polynesia and other islands of the Pacific in 2014. The limited evidence on ZIKAV before 2015 is not surprising—research funding is not prioritized for pathogens that are considered to be inconsequential. Since ZIKAV was introduced in the Americas between 2015 and 2016, more than 1,300 articles have been published on the subject, an increase of more than 1,000%. Yet, even with this impressive outpouring of evidence, we are still just scratching the surface in understanding the full impact this virus will have on the health of the people of this continent and in other parts of the world.

There are no easy solutions for the challenge posed by the limited resources available for tropical infectious disease research considering the growing list of emerging pathogens, concurrent with increasing vulnerabilities caused by climate change and climactic phenomena such as El Nino and La Nina. Therefore, we must rely on innovative approaches to guide a risk-based stratification of pathogens that are likely to cause significant epidemics. The WHO recently prioritized several pathogens likely to cause severe outbreaks for which there is urgent need to target interventions.^7^ Similarly, novel strategies such as virus genome sequencing and mathematical modeling have identified 37 viruses with some ability to spread between people but that have not yet been the cause of an epidemic.^8^ These efforts are good examples of marshaling both expertise and limited funding to expand current knowledge on transmission, prevention, and control of priority pathogens.

## Achieving the Vision of the International Health Regulations

Although the exact time and place of introduction of ZIKAV in the Americas is unknown, as early as November 2014 clusters of rash illness in Brazil's northeastern states were being observed.^1^ These clusters of rash illness appeared on the heels of the introduction into the Americas of chikungunya virus, another arbovirus carried by the same *Aedes* mosquito. Chikungunya had been introduced into the Caribbean just a year earlier, in December 2013, but spread very quickly through the region, with 1 million cases reported by the end of 2014. Chikungunya posed a new and significant public health challenge, emerging in Latin American and the Caribbean as the region was preparing for a possible introduction of Ebola virus, which was causing major disruptions in West Africa. The International Health Regulations (IHRs) obligate States Parties to develop core capacities to detect, assess, report, and respond to potential public health emergencies.^9^ The IHR themselves reflect a shared commitment to make investments in infrastructure and human resources to collectively respond expeditiously and effectively to public health events that might have international implications. Both the preparedness for Ebola and the response to chikungunya evidenced that although substantial progress had been made in implementing core capacities under the IHR, important weaknesses and limitations remained in the critical field of preparedness.^10^

As had happened with chikungunya, the identification of the cluster of rash illness caused by the ZIKAV was alerted by astute front-line health-care workers who first realized that they were detecting something unusual. Indeed, the ZIKAV experience proves once again that good clinical judgment and awareness of atypical events are crucial for the timely detection of emerging and reemerging diseases. It also points to the importance of investing in the health workforce as the first line of defense against infectious disease threats. On a regional level, good judgment and awareness are also required to discern signals generated by event-based surveillance, and to respond with appropriate and commensurate urgency in the absence of strong evidence. With this in mind, continued, long-term investment in enhancing public health infrastructure, with particular emphasis on strengthening and retaining the health-care workforce, is one of the most effective strategies in implementing early warning systems for the detection of acute infectious events.

## New Tools for Persistent Problems

There is a still long way to go on ZIKAV—the 50 years lead time provided since its original identification were not enough to prepare us for an outbreak of the magnitude of that observed in the Americas or for the complications brought with it. As ZIKAV disease was closely related to dengue and yellow fever, its circulation in Latin America and the Caribbean proved difficult to diagnose against a backdrop of immunity to other flaviviruses. Similarly, the available defenses against the mosquito responsible for the transmission of these viruses are no longer sufficient to resist its aggressive spread. Therefore, the development of affordable new tools by the scientific community, including diagnostic tests and a vaccine against ZIKAV, as well as innovation in vector control, are urgent priorities. Our health systems will need to be prepared to ensure such new tools are introduced and that their benefits reach everyone, not merely a few.

The WHO Blueprint for Action to Prevent Epidemics aims to accelerate research and development of innovative tools to combat emerging and reemerging infectious diseases.^7^ The recently created Coalition for Epidemic Preparedness Innovations intends to facilitate new partnership models that promote the development of new vaccines, diagnostics, and therapeutics to contain outbreaks of emerging infectious diseases.^11^ The potential success of these initiatives will rely on global coordination and collaboration in addressing shared challenges posed by emerging and reemerging infectious diseases.

## Final Considerations

Latin America and the Caribbean have approximately 500 million persons living in areas at risk for transmission of ZIKAV. The World Bank has estimated that the short-term economic impact of ZIKAV disease in the region of the Americas will be approximately US$3.5 billion.^12^ This estimate, however, is predicated on a swift, well-coordinated international response to the virus. Not included in these costs are the costs of managing a case of microcephaly throughout the life of a child. Furthermore, the impact of ZIKAV on families affected by microcephaly cannot be estimated. The full impact of ZIKAV on the central nervous system will only become apparent in the coming years, as children who were infected in utero fail to reach their developmental milestones. Furthermore, health systems will be challenged to care for children born with these complications for years to come.

New diseases bring new challenges, and decisions must be made even with limited evidence. The guiding principle in the case of ZIKAV in the Americas has been to act with caution but also with due urgency, using the best evidence available and placing priority on protecting the most vulnerable. The fight against ZIKAV must continue even if the current outbreak fades away. Future epidemic waves of ZIKAV, which will put additional people at risk, remain likely.
